# Free-water diffusion MRI detects structural alterations surrounding white matter hyperintensities in the early stage of cerebral small vessel disease

**DOI:** 10.1177/0271678X221093579

**Published:** 2022-04-11

**Authors:** Carola Mayer, Felix L Nägele, Marvin Petersen, Benedikt M Frey, Uta Hanning, Ofer Pasternak, Elina Petersen, Christian Gerloff, Götz Thomalla, Bastian Cheng

**Affiliations:** 1 Department of Neurology, University Medical Center Hamburg-Eppendorf, Hamburg, Germany; 2 Department of Diagnostic and Interventional Neuroradiology, University Medical Center Hamburg-Eppendorf, Hamburg, Germany; 3 Department of Psychiatry, Brigham and Women’s Hospital, Harvard Medical School, USA; 4 Department of Radiology, Brigham and Women’s Hospital, Harvard Medical School, USA; 5 Clinical for Cardiology, University Heart and Vascular Center, Germany; 6 Population Health Research Department, University Heart and Vascular Center, Hamburg, Germany

**Keywords:** Aging, brain imaging, cerebrovascular disease, diffusion weighted MRI, small vessel disease

## Abstract

In cerebral small vessel disease (CSVD), both white matter hyperintensities (WMH) of presumed vascular origin and the normal-appearing white matter (NAWM) contain microstructural brain alterations on diffusion-weighted MRI (DWI). Contamination of DWI-derived metrics by extracellular free-water can be corrected with free-water (FW) imaging. We investigated the alterations in FW and FW-corrected fractional anisotropy (FA-t) in WMH and surrounding tissue and their association with cerebrovascular risk factors. We analysed 1,000 MRI datasets from the Hamburg City Health Study. DWI was used to generate FW and FA-t maps. WMH masks were segmented on FLAIR and T1-weighted MRI and dilated repeatedly to create 8 NAWM masks representing increasing distance from WMH. Linear models were applied to compare FW and FA-t across WMH and NAWM masks and in association with cerebrovascular risk. Median age was 64 ± 14 years. FW and FA-t were altered 8 mm and 12 mm beyond WMH, respectively. Smoking was significantly associated with FW in NAWM (p = 0.008) and FA-t in WMH (p = 0.008) and in NAWM (p = 0.003) while diabetes and hypertension were not. Further research is necessary to examine whether FW and FA-t alterations in NAWM are predictors for developing WMH.

## Introduction

White matter hyperintensities of presumed vascular origin (WMH) are manifestations of cerebral small vessel disease (CSVD) on fluid-attenuated inversion recovery (FLAIR) magnetic resonance imaging (MRI) and are characterized by white matter axon loss, demyelination, and gliosis.^1^ They are particularly prevalent with increasing age and extent of cerebrovascular risk factors and are associated with cognitive impairment, dementia, stroke, depression, and mortality.^2–7^ However, in CSVD, microstructural alterations of the white matter are not confined to the visible WMH but also extend to brain areas without apparent changes on FLAIR, referred to as normal-appearing white matter (NAWM).^1^

Microstructural alterations of the white matter can be quantified with metrics derived from diffusion-weighted MRI (DWI). For conventional diffusion-tensor imaging (DTI) metrics, a tensor model is applied on DWI which calculates a single compartment of the diffusion signal within each voxel of the brain. However, tensors from a single compartment are susceptible for partial volume effects which occur when several tissue types reside in the same voxel. For example, the presence of extracellular free-water (FW) can contaminate the derived DTI metrics and limit the biological specificity of DTI.^8^ To overcome this limitation, an algorithm for FW elimination was developed which models two compartments of the diffusion signal. One compartment models FW as isotropic with diffusivity of water at body temperature. The voxel-wise fractional volume of the FW compartment is used to measure its contribution. The second compartment – the tissue compartment – applies a diffusion tensor to characterize diffusion in the proximity of cells, from which scalar measures such as fractional anisotropy of the tissue (FA-t) can be derived.^8^ When conventional and FW-corrected DTI metrics were compared, FW-corrected measures showed the strongest association with demographic parameters and cognitive functioning and was also superior in predicting mild cognitive impairments (MCI), Alzheimer’s disease and vascular dementia.^9–16^

In CSVD, the microstructural properties of NAWM are intricate. Specifically, conventional DTI metrics including fractional anisotropy (FA) and mean diffusivity (MD) indicate that the microstructural alterations of NAWM range from almost none to extensive, depending on factors such as age, WMH burden and cognitive functioning.^17–20^ In this work, we set to identify if FW captures microstructural changes of the white matter depending on the distance to WMH, defining larger areas surrounding visible WMH as ‘WMH penumbra’.^21^ We therefore examined FW and FA-t alterations in multiple NAWM regions that are adjacent to the WMH with different distances, to better identify the WMH penumbra. Moreover, we aimed to identify if FW and FA-t are associated with cerebrovascular risk factors in WMH and WMH penumbra.

## Materials and methods

### Study design and participants

We analysed data from participants of the Hamburg City Health Study (HCHS). HCHS is a prospective, single-centre, epidemiologic cohort study aiming to improve the understanding of risk factors and prognosis in major chronic diseases. A detailed description of the study design was published previously.^22^ In short, 45,000 citizens of Hamburg, Germany, between the age of 45 and 74 years are invited to an extensive baseline evaluation. Brain MRI is conducted in a randomly selected control group and in a subgroup of participants with increased risk for cardiovascular diseases as defined by a Framingham Risk Score >7.^23^ For the current analysis, we included the first 1,000 brain MRI datasets from HCHS participants at baseline. 21 participants had to be excluded because of missing imaging data (9 without imaging data, 3 without FLAIR, 9 without DWI), 39 participants because of incomplete DWI acquisition, 1 participant because of poor FLAIR image quality, and 9 participants because of technical issues during WMH segmentation. After initial image processing, 30 participants had segmented WMH <4 voxels and were therefore excluded. The ethics committee of the State of Hamburg Chamber of Medical Practitioners (Ethik-Kommission Landesärztekammer Hamburg, PV5131) approved the HCHS, and all participants provided written informed consent. The HCHS has been registered at ClinicalTrials.gov (NCT03934957). The HCHS design ensures that all involved individuals abide by the ethical principles described in the current revision of the Declaration of Helsinki, by Good Clinical Practice (GCP) and by Good Epidemiological Practice (GEP). The data underlying this article cannot be shared publicly for the privacy of individuals that participated in the study.

### Clinical data assessment

All participants of the HCHS received detailed anamnestic and clinical examination of which age, sex, (family) history of diseases and cardiovascular risk factors are of interest for the current study. A detailed study protocol has been published previously.^22^ In short, diabetes was defined as either self-reported prevalence of diabetes or a blood glucose level >126 mg/dl. Hypertension was defined as either self-reported prevalence of hypertension, blood pressure >=140/90 mmHg or intake of antihypertensive medication. The smoking status (yes/no) refers to active smokers and non-smokers.

#### MRI sequences

All images were acquired on a single 3T Siemens Skyra MRI scanner (Siemens, Erlangen, Germany) and applied the same imaging protocol. For 3D T1-weighted anatomical images, rapid acquisition gradient-echo sequence (MPRAGE) was used with the following sequence parameters: repetition time (TR) = 2500 ms, echo time (TE) = 2.12 ms, 256 axial slices, slice thickness (ST) = 0.94 mm, and in-plane resolution (IPR) = 0.83 × 0.83 mm. 3D T2-weighted FLAIR images were acquired with the following parameters: TR = 4700 ms, TE = 392 ms, 192 axial slices, ST = 0.9 mm, and IPR = 0.75 × 0.75 mm. For single-shell diffusion MRI, 75 axial slices were obtained covering the whole brain with gradients (b = 1000 s/mm^2^) applied along 64 noncollinear directions with the following sequence parameters: TR = 8500 ms, TE = 75 ms, ST = 2 mm, IPR = 2 × 2mm with an anterior-posterior phase-encoding direction.

### Imaging post-processing and definition of regions of interest

**Figure 1.**
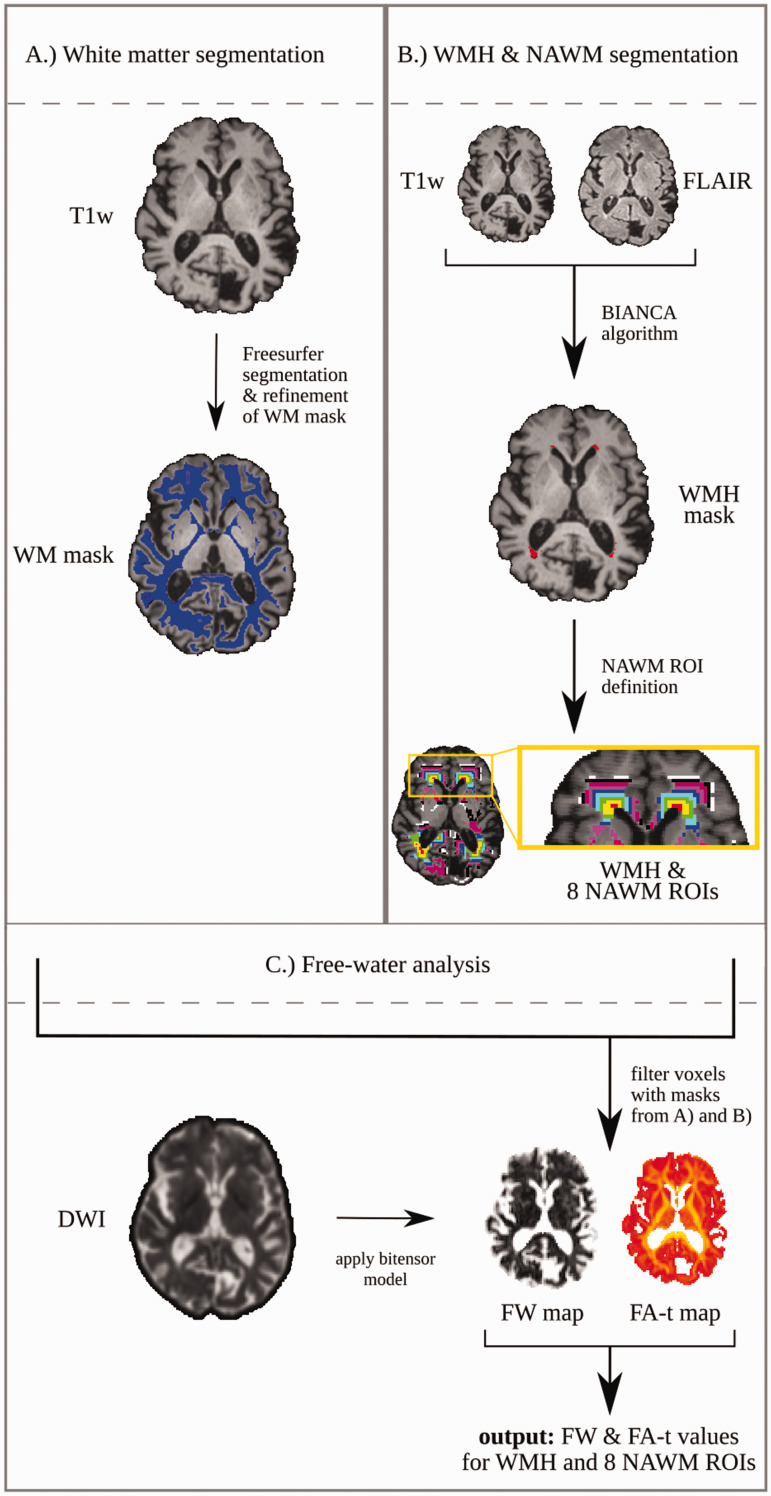
Visualization of the image processing pipeline. (a) Definition of white matter (WM) masks using T1-weighted images and Freesurfer.^24^ The WM mask was further refined by excluding structures such as brainstem and cerebellum. (b) T1-weighted images and FLAIR were used for segmentation of the white matter hyperintensities (WMH) with a trained automatic algorithm (BIANCA).^25^ The WMH mask was registered and resized to match the original DWI data resolution (2x2x2mm). Registered WMH masks were repeatedly dilated by one voxel and filtered with the white matter mask. (c) A bi-tensor model was applied on original DWI data to extract a tissue compartment and a free-water compartment from which free-water and free-water corrected FA (FA-t) maps were extracted. The WMH and NAWM ROIs were applied on the free-water and FA-t maps to calculate the average values for each region. DWI: diffusion-weighted image; FA: fractional anisotropy; FLAIR: fluid-attenuated inversion recovery; FW: free-water; NAWM: normal-appearing white matter; ROI: region of interest T1w: T1-weighted image; WM: white matter; WMH: white matter hyperintensities.

An overview of the image processing pipeline is visualized in Figure 1. FLAIR and T1-weighted images were resampled to 1 × 1 × 1 mm for the purpose of the registration between modalities. To register the structural images into DWI space without degrading the image resolution to the DWI (2 × 2 × 2 mm), we created a registration target (resampled DWI into 1 × 1 × 1 mm). The FLAIR and T1-weighted images were then non-linearly registered on the 1x1x1mm DWI with nearest-neighbour interpolation. Except for the WMH segmentation which happened on the 1 × 1 × 1 mm structural images, all subsequent steps of the imaging analysis were conducted on the original DWI in 2 × 2 × 2 mm resolution and the registered FLAIR and T1-weighted images in 1 × 1 × 1 mm resolution.

The standardized Freesurfer processing pipeline (Version 5.3) was applied on the registered T1-weighted images for the segmentation of brain structures including grey and white matter, ventricles, brainstem and cerebellum.^24^ Imaging data of insufficient quality for segmentation were excluded. For the calculation of the brain volume, the brain tissue without the ventricles was summed. White matter masks were further edited by subtracting the brainstem, cerebellum, and voxels located at the boundary to the grey matter (Figure 1(a)).

### Segmentation of WMH and NAWM ROIs

WMH were segmented with FSL’s Brain Intensity AbNormality Classification Algorithm (BIANCA).^25^ The fully automated, supervised k-nearest neighbour (k-NN) algorithm is based on native-space T1-weighted and FLAIR images and considers spatial information of each voxel after linear registration to Montreal Neurological Institute (MNI) space. As a training dataset for the k-NN algorithm, 100 FLAIR images were manually segmented by two experienced investigators (CM & MP) independently to form the training dataset (mean Dice Similarity Index of the training dataset: 0.63). Based on the segmentation of the automatic algorithm, the WMH volume was calculated. The WMH mask was registered into DWI-space by applying the warp fields from the structural image registration. After registration, the WMH mask was resized via interpolation and binarized to match the resolution of the original DWI data (2 × 2 × 2 mm) to enable overlay on FW and FA-t maps in the subsequent analyses.

After resizing of the WMH mask, we filtered them for minimum WMH size of 4 voxels (equivalent to 0.032 ml on a 2 × 2 × 2 mm image). By setting this threshold, we ensured that all imaging parameters measured within WMH are based on a representative number of voxels. Consequently, all participants with a WMH volume <0.032 ml were excluded. WMH load was defined as the ratio of WMH volume and brain volume. We further calculated the logarithm of the WMH load, referred to as ‘log WMH load’, to obtain normally distributed data. For the analysis of NAWM, regions of interest (ROIs) were created by multiple dilations in the 3-dimensional space in white matter areas adjacent to the WMH in 1 voxel increments (2 mm). This process was repeated 8 times, creating 8 NAWM masks representing increasing distance to the WMH. We avoided duplicate inclusion of voxels in more than one ROI by excluding all voxels overlapping with the previously created ROI in each dilation. In the final step, a total of 8 rim-shaped ROIs (each 2 mm size, in total a diameter of 16 mm surrounding WMH) were available and refined by including only voxels from the pre-defined white matter segmentation (Figure 1(b)). The size of the WMH penumbra was chosen based on previous literature.^26,27^

### Calculation of FW & FA-t

The bi-tensor model optimized for single-shell DWI was fitted to the original DWI data to calculate the signal attenuation for extracellular FW and tissue compartments separately.^8^ The regularized non-linear fit yielded FW maps, and FW-corrected diffusion tensors of the tissue compartment, from which the FW-corrected fractional anisotropy (FA-t) was calculated. Utilizing WMH and surrounding NAWM ROIs, FW and FA-t were extracted and subsequently averaged for each ROI for further statistical analysis (Figure 1(c)).

### Statistical analysis

Demographic data was summarized and reported with median and interquartile range (IQR). Normality of the data was checked with skewness and kurtosis, with the thresholds suggested for large datasets.^28^ We conducted two separate statistical analyses. First, we tested for differences of FW and FA-t between WMH and surrounding NAWM ROIs with increasing distance to the WMH. For this purpose, we conducted separate linear mixed-effects models with either FW or FA-t as the dependent variable. We added a variable with 9 categories (i.e., WMH and 8 NAWM ROIs) and pre-defined contrasts with forward difference coding to compare each white matter ROI with the next rim of NAWM ROI. Age, sex, and log WMH load were added as independent variables. Since FA-t and FW were measured at multiple regions within subjects, a random effect was included to control for individual differences. Based on the results from this model, NAWM ROIs with significantly different FW values were merged into one single ROI, termed ‘WMH-FW-penumbra’. In the second analysis, we tested the association of FW and FA-t values in WMH and WMH-FW-penumbra ROIs with cerebrovascular risk factors separately in two multivariate linear regressions. As before, age, sex, and log WMH load were added as independent variables next to variables of vascular risk (i.e., diabetes, smoking and hypertension). Results of all linear regressions are presented with standardized coefficients (β) and p-values (p). Effects were interpreted as significant if p < 0.05. All analysis was carried out using R Version 3.6.3.^29^

#### Supplementary analysis

To investigate the contribution of physiological, CSVD-independent effects on alterations of free-water in WMH-FW-penumbra, we conducted two post-hoc, supplementary analyses: 1) we separately calculated periventricular alterations of free-water in the WMH-FW-penumbra of deep and periventricular WMH. This was done to investigate if similar effects in the WMH-FW-penumbra would be observed in deep WMH, which occur in a much more heterogeneous spatial pattern as compared to periventricular WMH, which are more stereotypically located in the white matter in close proximity to the ventricles.^30,31^ 2) We compared free-water extent in the WMH-FW-penumbra in both participants from the first (lowest) and forth (highest) quartile of WMH volume. This approach was chosen to investigate if the extent of free-water changes would occur at similar distances from WMH independent of WMH extent. Methods and detailed results can be found in the supplement.

## Results

### Study sample characteristics

We included MRI data of the first N = 1,000 participants of the HCHS in our analysis. The final dataset comprises 900 participants (412 females; 45.8%) with a median age of 64 years (IQR = 14). The sample characteristics are summarized in Table 1.

**Table 1.** Sample characteristics with demographic data, cerebrovascular risk factors, and MRI measures.

Demographic characteristics	
Female sex, n (%)	412 (45.8%)
Age [years], median (IQR)	64 (14)
Cerebrovascular risk factors	
Active smoker, n (%)	141 (15.7%)
Diabetes mellitus,^a^ n (%)	73 (8.1%)
Hypertension,^b^ n (%)	618 (68.7%)
Conventional MRI measures	
Total brain volume [ml], median (IQR)	1,483 (203)
WMH volume [ml], median (IQR)	0.66 (1.4)
WMH load [%], median (IQR)	0.04 (0.1)

IQR: interquartile range; ml: millilitre; n: number of participants; WMH: white matter hyperintensities.

^a^Prevalence of diabetes mellitus was defined as blood glucose level >126 mg/dl or self-report.

^b^Prevalence of hypertension was defined as blood pressure >= 140/90 mm/Hg, intake of antihypertensive medication or self-report.

### Differences in white matter microstructure across ROIs

**Figure 2.**
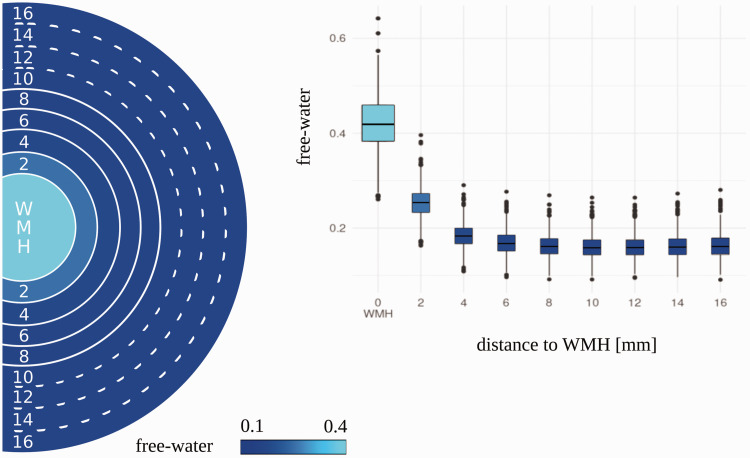
Distribution of free-water in white matter hyperintensities (WMH) and adjacent normal-appearing white matter regions. The boxplot displays the raw free-water distribution across the regions. The visualized circle displays the color-coded free-water in each region. A solid line between two adjacent regions represents a significant difference in free-water, based on the outcome of the multivariate linear regression. A dotted line represents no significant difference between the adjacent regions. mm: millimetre; WMH: white matter hyperintensities.

Results from linear mixed-effects models demonstrated significant differences in FW between concentrically adjacent ROIs including WMH and the first 4 NAWM ROIs (2 mm–8 mm) with the highest FW in the WMH and lower FW values with increasing distance from the WMH (Table 2, Figure 2). The differences between ROIs were significant after controlling for age, sex, and log WMH load. There were no significant differences of FW in adjacent ROIs between 10 mm–16 mm. Independent from the region, the results show that FW values were higher with increasing age (β = 0.008, p < 0.001) and higher log WMH load (β = 0.007, p < 0.001) while sex was not significant (β = 0.003, p = 0.051).

**Table 2.** Results of multivariate mixed-effects linear regression analysing free-water and FA-t in WMH and adjacent NAWM ROIs.

	Free-water		FA-t	
	β	p	β	p
Intercept	0.205	**<0.001**	0.456	**<0.001**
age	0.008	**<0.001**	–0.002	**0.01**
Sex–female	0.003	0.051	–0.001	0.35
log WMH load	0.007	**<0.001**	–0.004	**<0.001**
ROI contrasts
WMH – 2 mm	0.166	**<0.001**	–0.059	**<0.001**
2mm–4 mm	0.07	**<0.001**	–0.008	**<0.001**
4mm–6 mm	0.015	**<0.001**	0.019	**<0.001**
6mm–8 mm	0.006	**<0.001**	0.015	**<0.001**
8mm–10 mm	0.002	**0.017**	0.011	**<0.001**
10mm–12 mm	<–0.001	0.932	0.007	**<0.001**
12mm–14 mm	–0.001	0.154	0.004	**0.003**
14mm–16 mm	–0.001	0.18	0.002	0.141

Two multivariate linear regression models were conducted with either free-water or FA-t as the dependent variable, as indicated above. P-values <0.05 are indicated in bold. Models are additionally adjusted for random effects of individual differences.

β: standardized estimate; FA-t: free-water corrected fractional anisotropy; log: logarithmic; mm: millimetre; NAWM: normal-appearing white matter; p: p-value; ROI: region of interest; WMH: white matter hyperintensities.

**Figure 3.**
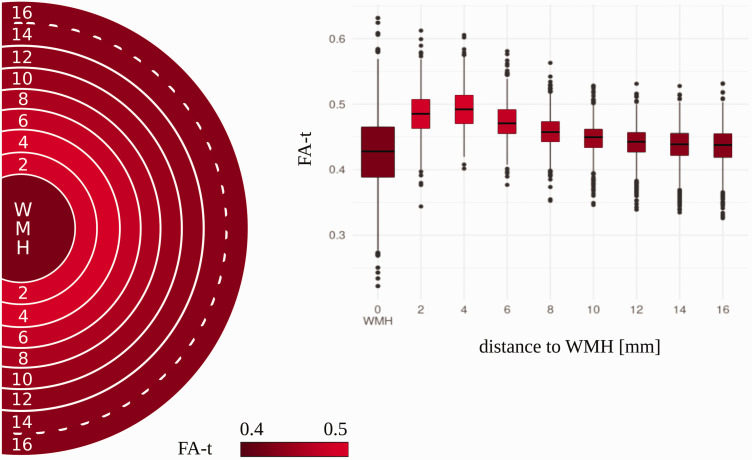
Distribution of free-water corrected fractional anisotropy (FA-t) in white matter hyperintensities (WMH) and adjacent normal-appearing white matter regions. The boxplot displays the raw FA-t distribution across the regions. The visualized circle displays the color-coded FA-t in each region. A solid line between two adjacent regions represents a significant difference in FA-t, based on the outcome of the multivariate linear regression. A dotted line represents no significant difference between the adjacent regions. FA-t: free-water corrected fractional anisotropy; mm: millimetre; WMH: white matter hyperintensities.

The linear mixed-effects model also revealed that FA-t differs significantly in all concentrically adjacent ROIs between WMH and 14 mm distance from the WMH. These differences were significant after controlling for age, sex, and log WMH load (Table 2, Figure 3). FA-t was the lowest in WMH, increased to a peak at the 4 mm ROI and declined with further increasing distance. Of all comparisons of concentrically adjacent ROIs, only the comparison of FA-t between the ROI at 14 mm with the ROI at 16 mm did not show a significant difference. Independent of the white matter region, FA-t was generally significantly lower with higher age (β = –0.002, p = 0.01) and higher log WMH load (β = –0.004, p < 0.001) while sex did not have a significant effect (β = –0.001, p = 0.35).

### Association of microstructural integrity with cerebrovascular risk factors

Based on the results of the ROI analysis, the ‘WMH-FW-penumbra’ was defined and included the first 4 NAWM ROIs (2 mm – 8 mm) where significantly increased FW was found between concentrically adjacent ROIs. In the second part of the analysis, we examined the association of FW and FA-t with cerebrovascular risk factors both in WMH and in the WMH-FW-penumbra. Results from multivariate linear regression demonstrated higher FW in WMH-FW-penumbra in active smokers (β = 0.006, p = 0.008) compared to non-smokers; no significant differences between smokers and non-smokers were found in WMH (β = 0.001, p = 0.793). Additional cerebrovascular risk factors were not significantly associated with FW. Moreover, FW in WMH and WMH-FW-penumbra was higher with increasing age (effect in WMH: β = 0.008, p < 0.001; effect in penumbra: β = 0.007, p < 0.001) and higher log WMH load (effect in WMH: β = 0.017, p < 0.001; effect in penumbra: β = 0.004, p < 0.001), next to a significant main effect of sex on FW in WMH (β = –0.008, p = 0.031). See Table 3 for all results of the linear regression.

**Table 3.** Results of multivariate linear regression models with cerebrovascular risk factors and free-water/FA-t in WMH and WMH-FW-penumbra.

	Free-water				FA-t			
	WMH		penumbra		WMH		penumbra	
	β	p	β	p	β	p	β	p
Intercept	0.424	**<0.001**	0.192	**<0.001**	0.431	**<0.001**	0.478	**<0.001**
age	0.008	**<0.001**	0.007	**<0.001**	0.008	**0.001**	–0.004	**<0.001**
sex – female	–0.008	**0.031**	–0.003	0.084	–0.011	**0.008**	–0.002	0.336
log WMH load	0.017	**<0.001**	0.004	**<0.001**	0.001	0.775	–0.003	**0.003**
smoking – active	0.001	0.793	0.006	**0.008**	–0.015	**0.008**	–0.007	**0.003**
diabetes – yes	–0.003	0.639	<–0.001	0.953	0.007	0.607	0.002	0.533
hypertension – yes	<0.001	0.997	0.002	0.227	0.002	0.316	0.003	0.212

The dependent variable is either free-water or FA-t measured in WMH and in WMH-FW-penumbra, as indicated in the table. The penumbra is defined as the normal-appearing white matter in the direct surrounding of WMH with higher free-water (diameter 8 mm). P-values < 0.05 are indicated in bold.

β: standardized estimate; FA-t: free-water corrected fractional anisotropy; log: logarithmic; p: p-value; WMH: white matter hyperintensities.

For FA-t, results of multivariate linear regression showed that active smokers had significantly lower FA-t values in WMH and WMH-FW-penumbra compared to non-smokers (effect in WMH: β = –0.015, p = 0.008; effect in penumbra: β = –0.007, p = 0.003). Other vascular risk factors, i.e., diabetes and hypertension, were not significantly associated with FA-t. Moreover, FA-t in both WMH and WMH penumbra was significantly depending on age (effect in WMH: β = 0.008, p = 0.001; effect in penumbra: β = –0.004, p < 0.001) next to significant main effects of sex on FA-t in WMH (β = –0.011, p = 0.008) and log WMH load on FA-t in WMH-FW-penumbra (β = –0.003, p = 0.003). See Table 3 for all results of the linear regression.

### Supplementary analysis

Results from our post-hoc analysis show that 1) both WMH in the periventricular and deep white matter have increased FW in the surrounding tissue, and that 2) the extent of FW increase in the surrounding tissue was present similarly both in the first and last quartile of the periventricular WMH volumes.

## Discussion

We examined the microstructural alterations of white matter in WMH and surrounding tissue in a large population-based cohort at increased risk for cerebrovascular diseases using DTI derived metrics of extracellular FW and FA-t. We further examined the association of FW and FW-corrected FA with major vascular risk factors both in WMH and in the WMH-FW-penumbra. Our results show that FW was significantly higher in the NAWM up to 8 mm surrounding the WMH with a distance-dependent decline. Additional, post-hoc analysis revealed that the FW alterations in the NAWM is present both in periventricular and deep WMH and independent from the degree of periventricular WMH volume, indicating an anatomically independent effect. The spatial relationship of FA-t values was more complex with the highest value located in NAWM of 4 mm distance to WMH and decreasing further distant. As a second finding, active smokers had significantly higher FW and lower FA-t in the WMH-FW-penumbra while diabetes and hypertension were not significantly associated. This study therefore helps to determine the dimension of microstructural deterioration in the penumbra of WMH based on FW measurements and to assess the relationship between FW in this penumbra and cerebrovascular risk factors.

In CSVD, the NAWM surrounding apparent WMH is affected by microstructural alterations not visible on FLAIR and was previously described as the ‘WMH penumbra’, characterized by a stronger tendency to convert to WMH.^21,32,33^ Several studies examined the extent of alterations in the white matter by analysing DWI and perfusion data, indicating that the degree of microstructural deterioration in NAWM increases in closer proximity to WMH.^20,21,26,34^ However, results show heterogeneity in defining the diameter of the WMH penumbra which depends on the imaging parameter used – it varies from a 4 mm penumbra defined by decreased FA and increased MD up to a 12 mm penumbra defined by CBF, and 20 mm for a penumbra defined by blood-brain barrier leakage.^20,21,27,34^ Despite inconsistent findings regarding the spatial extent of the WMH penumbra, all studies found that WMH represent the peak of microstructural degeneration in CSVD.^20,21,26,27,34^

In the current analysis, we characterized the extent of the WMH penumbra based on free-water imaging, reflecting the microstructural properties of the WMH and surrounding NAWM. In line with the notion of microstructural damage peaking in WMH, we found that WMH were characterized by the highest FW and the lowest FA-t values. In addition, there was a constant increase in FW with closer proximity to WMH starting in NAWM of 8 mm distance to WMH. Previous literature already indicates that FW in NAWM varies depending on the diagnosis of Alzheimer’s Disease, presence of MCI, and dementia severity.^11,35^ However, the spatial distribution of FW in NAWM was not previously examined. Increased extracellular FW may be an indicator for microvascular degeneration, demyelination and fibre loss which are pathological markers of CSVD.^36,37^ Neuroinflammatory processes and blood-brain-barrier leakage next to damage of the ependyma leading to cerebrospinal fluid entering the brain parenchyma are additional pathological mechanisms which could result in increases of the extracellular FW in WMH and NAWM.^38–40^ Taken together, the results of our study suggest that pathological white matter alterations implied in CSVD are detectable with free-water imaging in and, most importantly, beyond visible WMH. Longitudinal studies will help to delineate factors which influence the progression of the WMH-FW-penumbra.

Next to FW, FA-t demonstrated a more complex pattern in the NAWM ROIs with highest values in NAWM of 4 mm distance and decreasing in both closer and more distant ROIs. The counterintuitive results were already observed in other studies examining FW-uncorrected FA-values.^20,41^ The peak of FA-t values as a halo surrounding the WMH is a finding discordant to the linear decrease of FW. A possible explanation for the peak in the direct surrounding of WMH is the majority of WMH in this study being located in the caps of the ventricles which contain densely bundles white matter tracts and, subsequently, have by nature higher FA-t values compared to white matter with a low density of tracts.^30,42,43^ Consequently, FA-t (or FA in general) is prone to local differences by higher values in regions with densely bundles white matter tract compared to regions outside the main fibre tracts. This bias can be unmasked with free-water imaging. Previous studies already found that FW is more correlated with clinical deficits and provides a greater biological specificity than conventional DTI metrics, where alterations in FA and MD can be explained by increases in FW.^8–10,12^

We examined the association of FW and FA-t with cerebrovascular risk factors represented by hypertension, smoking, and diabetes. Our results show a significant association of FW with smoking in the WMH-FW-penumbra. However, neither the relationship between smoking status and FW in WMH was significant nor was FW associated with other vascular risk factors. For FA-t, active smoking status was significantly associated with lower FA-t values in both WMH and the WMH-FW-penumbra, while diabetes and hypertension were not significantly associated. The results are in line with previous literature showing that chronic smokers have significantly lower FA and smoking cessation is positively related to lower FA in NAWM, but no study examined the effect of smoking on changes of FW in the NAWM so far.^44–46^ As a potential pathophysiological mechanism underlying the association with FW, chemical constituents of the cigarette smoke are suggested to cause neurotoxic swelling of the brain and plasma fluid leaking into the interstitial space which leads to more extracellular water.^47,48^ Previous literature also indicates an association of diabetes and hypertension with uncorrected FA in the brain white matter but their association with FW has not been examined previously.^46,49–51^ Our analysis revealed that FA-t and FW were not significantly associated with diabetes or hypertension. As an important limitation, we did not consider the time since diagnosis or differences between participants with or without antihypertensive medication - two factors which were shown to be associated with WMH severity.^3,52^ Additional analysis is necessary to examine whether diabetes and hypertension might have an influence on FW in a cohort with severe manifestations of CSVD, syndromes of neurodegeneration or other neurological diseases affecting the brain microstructure.

Generally, all significant effects reported in this paper were found in a cohort which displayed a relatively low degree of CSVD manifestation on MRI. Therefore, our study provides novel findings for early stages of CSVD demonstrating subtle alterations in the cerebral white matter surrounding WMH. Due to the selection of the participants from a population-based study, we cannot exclude an impact of physiological white matter microstructure on our findings. However, given the results from our post-hoc analysis, we would argue that our findings nonetheless indicate pathophysiological processes occurring in CSVD as a spectrum of increasing alterations of the white matter. The cross-sectional nature of our study is an additional limitation and further studies are needed to investigate the course of deterioration longitudinally and to examine whether increased FW is a predictor of conversion into WMH. Moreover, given the selection of participants from a population-based study, it is not possible to provide an a-priori categorization of unaffected individuals. While our results are based on the application of a bi-tensor model which accounts for signal contribution from tissue and extracellular, i.e., FW, compartments, it has been shown that perfusion significantly affects the estimation of the FW fraction.^53^ Therefore, novel three-compartment diffusion MRI methods which account for the signal contribution of blood perfusion, in addition to that of FW and tissue compartments, might be helpful to delineate the complex interplay of vascular and tissue-changes observed in CSVD.^53^ Such models require the acquisition of multi-shell diffusion data, for which more robust estimation of FW is also available.^54^

To conclude, we presented results of the, so far, largest free-water imaging analysis in a population-based study in the context of CSVD. We showed that the WMH-FW-penumbra has a size of 8 mm surrounding WMH and that FW increases in closer proximity to WMH. In contrast to that, FA-t shows a more complex spatial distribution to WMH with peaks at 4 mm distance which is congruent with previous studies. In addition, our analysis found significantly higher FW and lower FA-t in active smokers compared to non-smokers. Further research will be necessary to better understand the longitudinal trajectory of FW and cellular tissue alterations in NAWM and their spatial relationship to WMH. This might ultimately help to identify which factors are beneficial in preventing NAWM from the progression to WMH.

## Supplemental Material

sj-pdf-1-jcb-10.1177_0271678X221093579 - Supplemental material for Free-water diffusion MRI detects structural alterations surrounding white matter hyperintensities in the early stage of cerebral small vessel disease Click here for additional data file. Supplemental material, sj-pdf-1-jcb-10.1177_0271678X221093579 for Free-water diffusion MRI detects structural alterations surrounding white matter hyperintensities in the early stage of cerebral small vessel disease by Carola Mayer, Felix L Nägele, Marvin Petersen, Benedikt M Frey, Uta Hanning, Ofer Pasternak, Elina Petersen, Christian Gerloff, Götz Thomalla and Bastian Cheng in Journal of Cerebral Blood Flow & Metabolism
