# Supplementary material for: Free-water diffusion MRI detects structural alterations surrounding white matter hyperintensities in the early stage of cerebral small vessel disease
Source: J Cereb Blood Flow Metab. 2022 Apr 11;42(9):1707–18. doi: 10.1177/0271678X221093579 (PMC9441727; doi:10.1177/0271678X221093579)
Supplement: sj-pdf-1-jcb-10.1177_0271678X221093579 - Supplemental material for Free-water diffusion MRI detects structural alterations surrounding white matter hyperintensities in the early stage of cerebral small vessel disease [file sj-pdf-1-jcb-10.1177_0271678X221093579.pdf]

Supplementary Material

Free-water diffusion MRI detects structural alterations surrounding white matter hyperintensities in the early stage of cerebral small vessel disease

Carola Mayer[1], Felix L Nägele[1], Marvin Petersen[1], Benedikt M Frey[1], Uta Hanning[2], Ofer Pasternak[3,4], Elina Petersen[5,6], Christian Gerloff[1], Götz Thomalla[1] and Bastian Cheng[1]

[1] Department of Neurology, University Medical Center Hamburg-Eppendorf, Germany

[2] Department of Diagnostic and Interventional Neuroradiology, University Medical Center Hamburg-Eppendorf, Germany

[3] Department of Psychiatry, Brigham and Women's Hospital, Harvard Medical School, United States

[4] Department of Radiology, Brigham and Women's Hospital, Harvard Medical School, United States

[5] Clinical for Cardiology, University Heart and Vascular Center, Germany

[6] Population Health Research Department, University Heart and Vascular Center, Germany

Corresponding author

Carola Mayer, Department of Neurology, University Medical Center Hamburg-Eppendorf, Martinistraße 52, 20246 Hamburg, Germany. Email: c.mayer@uke.de

Supplement Material S1. Analysis of penumbral tissue for periventricular and deep WMH

Methods

In this additional analysis, we measured free-water (FW) measurements in the WMH and penumbra of periventricular and deep WMH separately to investigate if similar effects in the WMH penumbra would be observed in deep WMH, which occur in a much more heterogeneous spatial pattern as compared to periventricular WMH, which are confined to the white matter in close proximity to the ventricles.[1,2] To differentiate between periventricular and deep WMH, we applied a threshold of 10mm from the ventricles, as proposed previously.[1] After the differentiation, we applied the same analysis steps as in the main manuscript, separately for the deep WMH and periventricular WMH. Regions of interest (ROIs) were created by dilating the periventricular / deep WMH mask in the 3-dimensional space in 1 voxel increments (2mm). This process was repeated 8 times to create 8 normal-appearing white matter (NAWM) masks. To avoid confounding effects of periventricular WMH located in the penumbral tissue of deep WMH and vice versa, we excluded both the WMH mask and the first penumbral tissue mask (2mm surrounding WMH) of each WMH type from the other WMH type. Then, FW was extracted and averaged for each ROI.

The statistical analysis contained the same linear mixed-effects models as applied in the main manuscript, with FW as the dependent variable. Models were defined separately for periventricular and deep WMH.

Results

The linear mixed-effects models showed, that both WMH in the periventricular and deep white matter have increased FW in the surrounding tissue (table 1). For a visualization of the distribution of the deep and periventricular WMH, see figure 1. Although the effect is less extensive in the case of deep WMH (FW increased in up to 4mm distance to the lesion), the significant results both in periventricular and deep WMH indicate that FW alterations are not solely associated with the distance to the ventricles. Additionally, we see that the standardized effect sizes (β) decrease with increasing distance to both periventricular and deep WMH, indicating a direct relationship between FW in the penumbra and WMH.

Figure 1. Heatmap of the periventricular and deep WMH of the study sample (N = 900).

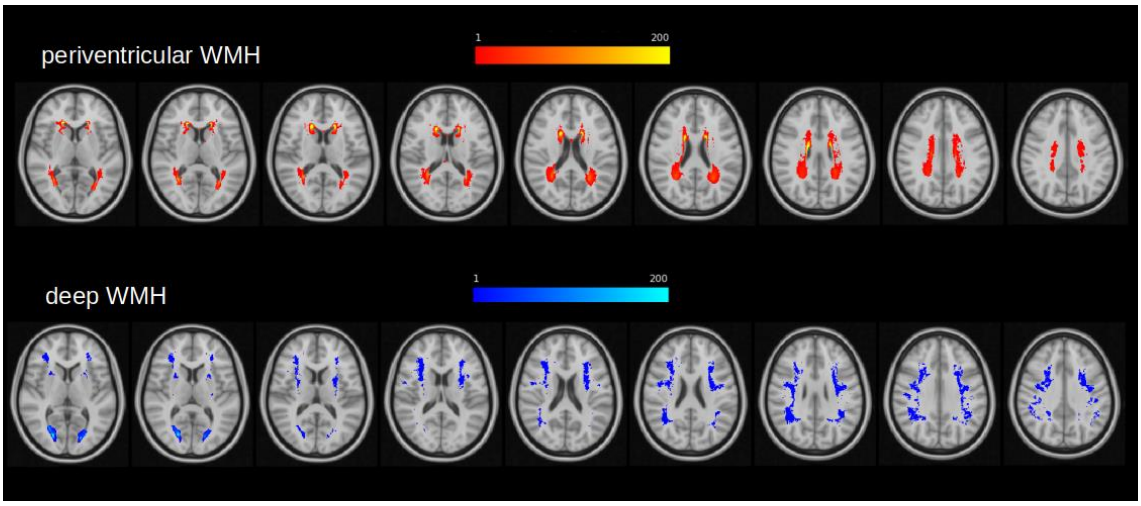

**Table 1.** Results of the linear mixed-effects models analyzing the free-water content in the deep and periventricular WMH.					
		Free-water in periventricular WMH		Free-water in deep WMH	
		β	p	β	p
Intercept		0.212	**<0.001**	0.197	**<0.001**
age		0.008	**<0.001**	0.005	**<0.001**
sex - female		-0.003	0.056	-0.006	**0.009**
log WMH load		0.008	**<0.001**	0.009	**<0.001**
ROI contrasts	WMH – 2mm	0.175	**<0.001**	0.119	**<0.001**
	2mm – 4mm	0.07	**<0.001**	0.052	**<0.001**
	4mm – 6mm	0.016	**<0.001**	0.011	**<0.001**
	6mm – 8mm	0.007	**<0.001**	< 0.001	0.623
	8mm – 10mm	0.004	**0.001**	-0.001	0.433
	10mm – 12mm	< 0.001	0.392	-0.001	0.31
	12mm – 14mm	< -0.001	0.7	-0.002	0.174
	14mm – 16mm	< -0.001	0.461	-0.002	0.248

Two multivariate linear regression models were conducted with free-water as the dependent variable. As indicated above, free-water in either the periventricular or deep WMH were included. P-values < 0.05 are indicated in bold. Models are additionally adjusted for random effects of individual differences.

Abbreviations: β = standardized estimate, log = logarithmic, mm = millimetre, p = p-value, ROI = region of interest, WMH = white matter hyperintensities.

Supplement Material S2. Analysis of penumbral tissue for lowest and highest quartile of periventricular WMH volume

Methods

As a second approach, we examined FW in the penumbra of periventricular WMH separately for participants in the first (<25%, Q1) and last (>75%, Q4) quartile of WMH volumes. For this, we divided the cohort into four quartiles based on their periventricular WMH volume and only considered the lowest and highest quartile for further analysis. Mean volumes for periventricular WMH were 0.08ml (IQR = 0.073) for Q1 and 3.4ml (IQR = 2.55) for Q4. A heatmap of the periventricular WMH volumes in both groups (Q1/Q4) is visualized in figure 2.

Results

Again, we applied two linear mixed-effects models as described in the main manuscript. In this analysis, only FW in the WMH and penumbral tissue of either Q1 or Q4 of the periventricular WMH was considered. The results are shown in table 2. In short, although the groups varied in periventricular WMH volume (i.e. the extent of WMH into periventricular white matter), the extent of FW increase in the WMH penumbra was similar for WMH in Q1 and Q4. The results indicate that the FW increases in proximity to WMH do not solely reflect the underlying, physiological anatomical structure.

Figure 2. Heatmap of the lowest and highest quartile of periventricular WMH volume in the study sample (N = 900).

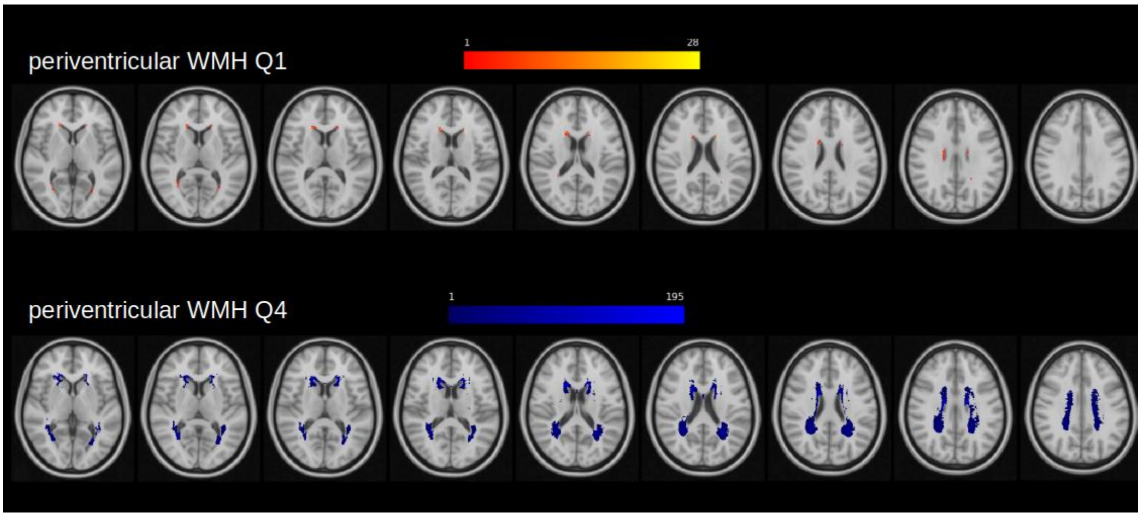

Table 2. Results of the linear mixed-effects models analyzing the free-water content in the first and fourth quartile of the periventricular WMH volume.

		Free-water in Q1 periventricular WMH volume		Free-water in Q4 periventricular WMH volume	
		β	p	β	p
Intercept		0.189	**<0.001**	0.222	**<0.001**
age		0.005	**<0.001**	0.006	**<0.001**
sex - female		< -0.001	0.962	-0.009	**0.008**
log WMH load		0.001	0.657	0.006	**<0.001**
ROI contrasts	WMH – 2mm	0.142	**<0.001**	0.183	**<0.001**
	2mm – 4mm	0.069	**<0.001**	0.069	**<0.001**
	4mm – 6mm	0.017	**<0.001**	0.013	**<0.001**
	6mm – 8mm	0.007	**0.001**	0.005	**0.001**
	8mm – 10mm	0.003	0.208	0.001	0.452
	10mm – 12mm	0.001	0.724	-0.002	0.295
	12mm – 14mm	< 0.001	0.916	-0.003	**0.031**
	14mm – 16mm	< 0.001	0.893	-0.003	0.061

Two multivariate linear regression models were conducted with free-water as the dependent variable. As indicated above, either participants in the first or fourth quartile of the periventricular WMH volume were included. P-values < 0.05 are indicated in bold. Models are additionally adjusted for random effects of individual differences.

Abbreviations: β = standardized estimate, log = logarithmic, mm = millimetre, p = p-value, Q1= first quartile (<25%), Q4 = fourth quartile (>75%), ROI = region of interest, WMH = white matter hyperintensities.

References

1. Griffanti L, Jenkinson M, Suri S, et al. Classification and characterization of periventricular and deep white matter hyperintensities on MRI: A study in older adults. *Neuroimage* 2018; 170: 174–181.

2. Kim KW, MacFall JR, Payne ME. Classification of White Matter Lesions on Magnetic Resonance Imaging in Elderly Persons. *Biol Psychiatry* 2008; 64: 273–280.
